# Microbiome and related structural features of Earth’s most archaic plant indicate early plant symbiosis attributes

**DOI:** 10.1038/s41598-022-10186-z

**Published:** 2022-04-20

**Authors:** Anchittha Satjarak, G. Karen Golinski, Marie T. Trest, Linda E. Graham

**Affiliations:** 1 Plants of Thailand Research Unit, Department of Botany, Faculty of Science, Chulalongkorn University, Bangkok, Thailand; 2 University of British Columbia Herbarium, Beaty Biodiversity Museum, University of British Columbia, Vancouver, BC Canada; 3 Department of Botany, Smithsonian National Museum of Natural History, Washington, DC USA; 4 Department of Botany, University of Wisconsin-Madison, Madison, WI USA

**Keywords:** Evolution, Plant sciences

## Abstract

Origin of earliest land plants from ancestral algae dramatically accelerated the evolution of Earth’s terrestrial ecosystems, in which microbial symbioses have played key roles. Recent molecular diversification analyses identify the rare, geographically-limited moss *Takakia* as Earth’s most archaic modern land plant. Despite occupying a phylogenetic position pivotal for understanding earliest plants, *Takakia* microbial associations are poorly known. Here, we describe symbiosis-related structural features and contig-based metagenomic data that illuminate the evolutionary transition from streptophyte algae to early embryophytes. We observed that *T. lepidozioides* shares with streptophyte algae secretion of microbe-harboring mucilage and bacterial taxa such as *Rhizobium* and genes indicating nitrogen fixation. We find that *Takaki*a root-analogs produce lateral mucilage organs that are more complex than generally understood, having structural analogies to angiosperm lateral roots adapted for N-fixation symbioses, including presence of intracellular microbes. We also find structural and metagenomic evidence for mycorrhiza-like species of glomalean fungi (including *Rhizophagus irregularis*) not previously known for mosses, as well as ascomycete fungi (e.g. *Rhizoscyphus ericae*) that associate with other early-diverging plants. Because *Takakia* is the oldest known modern plant genus, this study of plants of a remote locale not strongly influenced by human activities may indicate microbiome features of early land plants.

## Introduction

Two modern clades of land-adapted plants–bryophytes and vascular plants–arose from ancestral streptophyte green algae, with consequent dramatic ecological impacts on Earth’s terrestrial ecosystems^1^. The ability of early embryophytes to thrive on land has often been linked to microbial symbioses that improved access to growth-limiting minerals such as P and N and increased stress tolerance, illustrated by the ubiquity of modern beneficial symbioses^2^. Efforts to understand the evolutionary history of plant–microbe associations include 16S RNA gene marker studies indicating bacterial components of the microbiota^3^, use of multiple taxonomic markers derived from metagenomic sequence to infer prokaryotic and eukaryotic microbiome components^4^, or analyses of symbiosis-associated genes^5,6^ of modern streptophyte lineages, employing bryophytes to represent early plants. Recent molecular diversification studies indicate that the moss *Takakia* occupies a pivotal phylogenetic position as Earth’s oldest (most archaic) extant plant lineage, branching close to the divergence of bryophytes from vascular plants^1^. Although *Takakia* is known to possess particular genetic elements associated with microbial symbioses^5^, its microbiota are poorly studied, reflecting plant rarity and remote habitats. Knowledge of *Takakia* microbiota has largely been limited to evidence from microscopy that fungi occur within mucilage produced by plants collected from coastal British Columbia for structural analysis^7^, a drawing showing intracellular aseptate fungi in plants from the same locale^8^, and isolation of new bacterial genera from plants collected from a Tibetan glacier^9,10^.

To better illuminate the evolutionary history of plant-microbial associations, we conducted metagenomic and symbiosis-related structural analyses of *Takakia lepidozioides* plants collected from remote Haida Gwaii (formerly Queen Charlotte Islands), British Columbia, Canada, that were carefully separated from other plants and repeatedly washed to remove loosely-associated materials. Contigs assembled from metagenomic data were used for taxonomic assessments to improve identification confidence, because matches occurred over longer sequence regions than was the case for short reads more commonly employed^11^. Results were compared to metagenomic and symbiosis-related structural features we had previously described for other bryophytes and streptophyte algae that had been sampled, processed, and analyzed similarly, to facilitate evolutionary contrasts^12–15^.

Our discoveries include finding that *T. lepidozioides* shares with streptophyte algae cells that secrete microbe-harboring mucilage, and associated bacteria and genes indicating nitrogen fixation capacity. Microscopic and metagenomic evidence for species of mycorrhiza-like glomalean and ascomycete fungi not previously known for mosses suggest additional symbiosis features that might have characterized early land plants prior to the divergence of bryophytes from vascular plants.

## Results and discussion

### Metagenomic data confirm taxonomic identity of the plant host

Identification of the host moss as *Takakia lepidozioide*s S. Hatt & Inoue was supported by a > 1500 bp contig of 28S rDNA having 100% sequence identity to database reference sequence (Supplementary Table 1), > 2000 18S and > 5000 28S unassembled rDNA (rRNA gene) reads classifying as *T. lepidozioides* (Supplementary Tables 3, 4), and a full plastid genome (Supplementary Fig. 1) similar to one for this moss species that had been deposited in a database (NC_028738.1). The *T. lepidozioides* plastid genome we assembled from metagenomic data occurred in a single contig and displayed quadripartite structure; size 149,358 bp; 85 unique protein-coding genes; 33 unique tRNA genes; and 3 unique rRNA genes. By comparison to NC_028738.1, our reconstructed plastid genome was 243 bp longer; in the aligned region two sequences corresponded to one syntenic region with 99.7% identity. Polymorphisms, SNP and indels were present across the whole plastid genome in genic, intragenic, and intergenic regions.

### Host structures that facilitate microbial symbioses are more complex than previously understood

**Figure 1:**
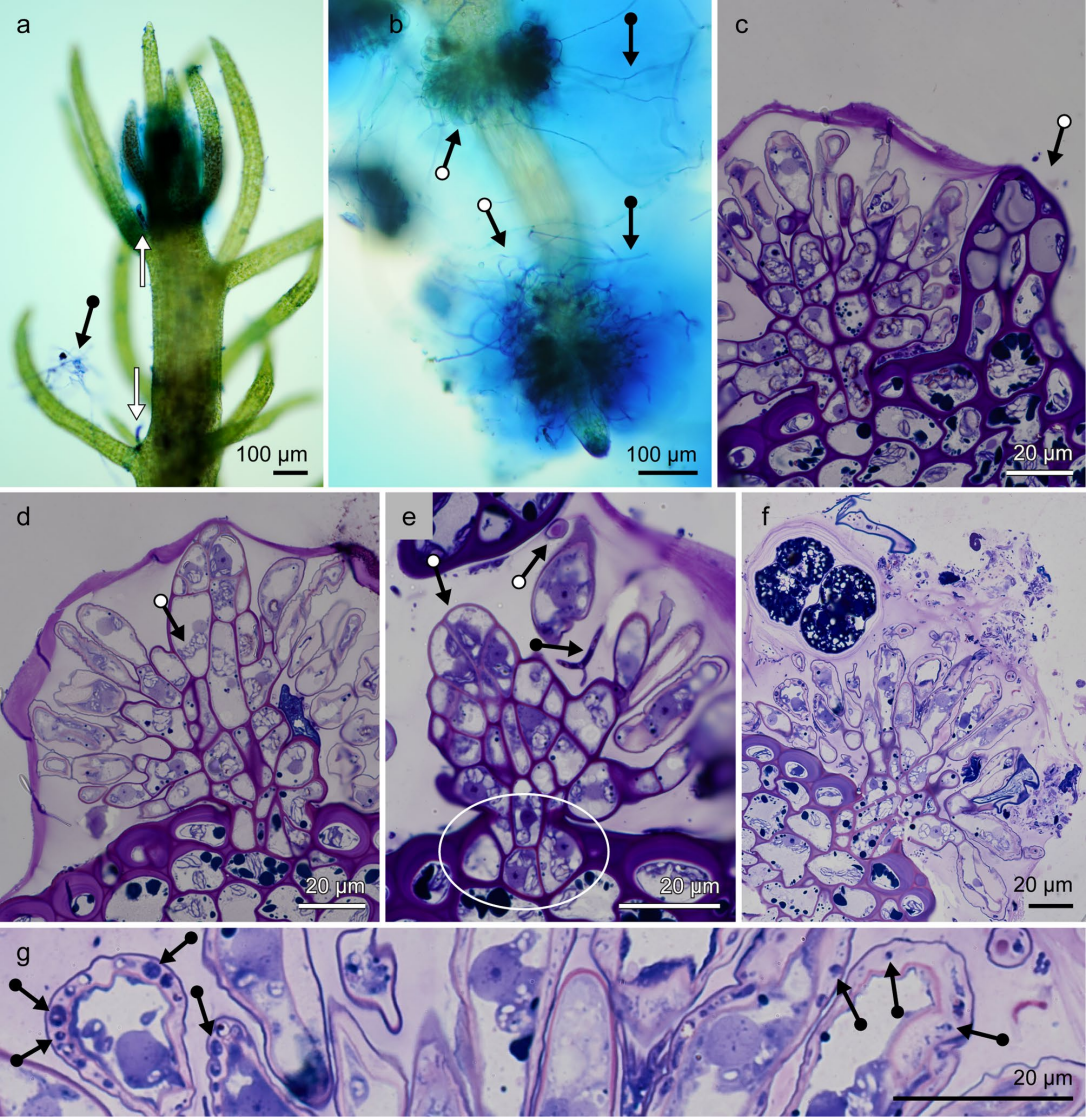
Structural evidence for *Takakia* mucilage organs that foster microbial associations. (**a**) Erect green axis with methylene blue-staining of apical tissues and short sub-apical filaments near phyllid axils (white arrows), and nearby surface mucilage harboring fungal hyphae (black arrow). (**b**), Pale rhizomatous axis showing methylene blue-stained apical tissue, a subapical collar-like array of mucilage-producing cells and additional mucilage-producing structures (white dot arrows) and associated mucilage clouds that include microbes (black dot arrows). (**c**), Toluidine blue-stained semi-thin section of a young developmental stage of the rhizomatous lateral mucilage organ, subtended by a small phyllid (arrow). Outer periphery of the mucilage cloud is conspicuously stained (top). Epidermal cell walls of the rhizomatous axis and phyllid are noticeably thickened and deeply-stained by comparison to cell walls of the mucilage organ, which are thinner and less deeply-stained. (**d**), Toluidine blue-stained semi-thin medial section of the parenchymatous core (arrow) of a young lateral rhizomatous mucilage organ, with apical meristem near the edge of the mucilage cloud. From the parenchymatous core emerges a hemispherical array of branched-filaments bearing mucilage cells whose tips are open at maturity, and from which mucilage is extruded. (**e**), Enlarged view of the parenchymatous core of a young lateral mucilage organ, showing the parenchymatous base (oval), whose cell walls are more similar in thickness and staining properties to those of the cortex of the rhizomatous axis than to its epidermal cells. Apical meristematic region of the lateral organ and cross-sectional view of the narrow end of a mucilage cell (white dot arrows), and branched fungal hypha within the mucilage cloud (black dot arrow). (**f**), Abundant microbes of diverse morphotypes in the mucilage cloud produced by a mature mucilage organ of *T. lepidozioides* viewed in toluidine blue-stained semi-thin section. Mucilage cells at the periphery of the mucilage organ contain pink-stained mucilage. Structures of sizes and shapes consistent with interpretation as microbes are commonly present in mucilage-producing cells, having likely entered through their open tips. (**g**), Higher magnification of a portion of part f, focusing on microbes occupying intracellular mucilage (arrows).

Consistent with previous observations of this moss species^7^, the body consisted of an erect 0.5–1 cm tall green axis bearing cylindrical leaf-like phyllids and longer, pale, horizontally- or vertically-oriented branching rhizomatous axes (also called stolons) proposed to be functionally analogous to roots^16^. Methylene blue (MB) staining indicated mucilage production by cells near apices of both erect and rhizomatous axes (Fig. 1 a,b), a feature that in other bryophytes has been proposed to protect meristems from drying^17^. A collar of mucilage-producing cells that we often observed to occur near the rhizomatous apex (see Fig. 1b), not previously described, might also facilitate growth through substrata, analogous to mucilage function at vascular plant root tips.

Methylene-blue-staining also characterized subapical epidermal outgrowths that secrete mucilage, the mucilage itself, and associated microbiota such as fungal hyphae (see Fig. 1 a,b), consistent with observations of the same species by others^7^. Our plants were also consistent in producing mucilage-secreting cells of two structural types, those having rounded ends that do not open, and those having narrow open tips at maturity, previously termed “beaked mucilage cells,” into which microbes have been observed to grow^7^. Here, we refer to such cells as open-tip mucilage cells. The closed-end mucilage cell type occurred at the ends of uniseriate, few-celled, unbranched filaments sometimes occurring near axils of leaf-like phyllids on erect green shoots, associated with extracellular mucilage thought to be secreted through cell walls^7^ (Fig. 1a). Erect green shoots also produced small clusters of open-tip mucilage cells at the ends of short branched filaments extending from the epidermis, as previously reported^18^.

By contrast, rhizomatous shoots displayed much larger, often hemispherical, arrays of open-tip mucilage cells, generating conspicuous mucilage clouds containing microbes (see Fig. 1b). By use of semi-thin, toluidine blue-stained sections we discovered that these larger rhizomatous arrays of mucilage cells are considerably more structurally-complex than smaller clusters whose development was previously reported^18^, and analogous in some ways to higher plant lateral roots that are specialized in ways that foster microbial symbioses. Each larger mucilage cell array consists of branched filaments bearing open-tip mucilage cells that arise on a core parenchymatous axis having an apical meristem (Fig. 1c,d), features indicating that these axes are lateral organs. We refer to these axes as mucilage organs. At least some mucilage organs emerged near a small phyllid having thickened, densely-staining cell walls similar to those of epidermal cells of the rhizomatous axis (Fig. 1c). By contrast, walls of cells at the parenchymatous bases and cores of mucilage organs were similar in thickness and staining to cell walls in the cortex of the rhizomatous (root-analog) axis, indicating endogenous origin (Fig. 1c-f). Cell walls of open-tip mucilage cells were conspicuously thin (see Fig. 1c,d) a feature that may aid the formation of open tips. Mucilage organ cells, though not green, contained at least one amyloplast, as did cells of the rhizomatous axis.

Semi-thin sections of mature mucilage organs reveal large populations of microbes of diverse morphotypes in the mucilage cloud (Fig. 1f), and also show evidence for presence of microbes within mucilage inside many of the host plant mucilage cells, such microbes having presumably entered via open tips (Fig. 1g). Some structural aspects of the *Takakia* mucilage organ (endogenous origin, apical meristem, and intracellular microbes) suggest analogies to features of angiosperm root nodules, modified lateral roots that support the N-fixation activities of rhizobia. Specialized lateral roots of a maize land race that produce conspicuous mucilage harboring N-fixing bacteria^19^ represent another angiosperm analogy to *Takakia* mucilage organs.

By contrast to other types of *Takakia* mucilage secretion structures (apical cells, uniseriate short filaments, small clusters of open-tip mucilage cells arising from branched epidermal filaments), the newly-described arborescent mucilage organs greatly amplify mucilage production, allowing increased capacity to harbor microbial associates. Though mucilage-production is known for other bryophytes, specialized cells having narrow tips that burst at mucilage release are uncommon. Homologous features occur among the later-diverging lineages of streptophyte algae, species of which produce extensive surface mucilage that harbors diverse microbiota, including nitrogen-fixing bacteria and fungi. Such algae include zygnemataleans, whose cell walls are sometimes penetrated by numerous pores through which mucilage is secreted^12,13^ and coleochaetaleans whose cells produce microbe-inhabiting mucilage clouds^14^ and that also produce narrow-tip cells that can produce exudates^20^. Such similarities suggest that mucilage-producing cells of *Takakia* reflect symbiosis-related traits of streptophyte algal ancestors.

Additional features shared with streptophyte algae include cell wall traits that may reduce potential for host harm by surface microbial biofilms. Overall *T. lepidozioides* body structure was resistant to hydrolysis, maintaining normal morphology even after extended treatment with concentrated acids. Fluorescence microscopy indicated that epidermal cell walls have autofluorescence features (yellow in violet excitation, blue-white in UV excitation) indicating presence of phenolic compounds associated with hydrolysis-resistance by streptophyte algae^21^. Presence of phenolic-rich, hydrolysis-resistant cell walls has also been proposed to explain fossil remains attributed to Sphagnales^22^, the moss lineage most closely-related to *Takakia*^1^, and so might likewise foster *Takakia* persistence in sediments long enough to form fossil remains. This new information indicating hydrolysis-resistance justifies efforts to search for fossils of *Takakia*-like early land plants.

### *Takakia lepidozioides* hosts mycorrhiza-like fungal associations

**Figure 2:**
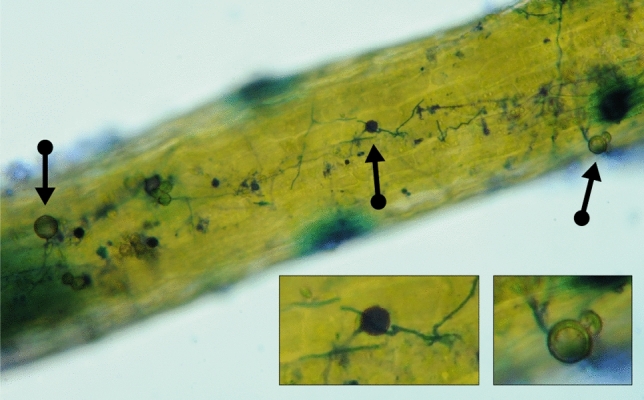
Structural evidence for mycorrhiza-like fungal associations. Host axis stained with methylene blue, showing structures having sizes and shapes consistent with interpretation as glomalean fungi. Aerial septate hyphae produce spherical spores of different developmental stages (arrows) and associated aseptate hyphae appear to occur beneath epidermal cell walls, forming blue-stained areas of sizes and shapes consistent with interpretation as finely-branched arbuscules (inset).

Methylene blue-stained branched structures having morphology and dimensions consistent with fungal hyphae that we observed were previously noted to occur within mucilage produced by both erect and rhizomatous axes, sometimes penetrating open-tip mucilage cells^7^. Not previously reported is our observation that epidermal cells of the erect green axes are associated with fungi having distinctive large, round spores (up to 20 µm diameter) borne on branches of robust aseptate hyphae (Fig. 2), features distinctive for glomalean fungi. The glomalean-like hyphae appeared to extend beneath host outer epidermal cell walls, producing intracellular structures consistent in size and location (Fig. 2 insert) with a published drawing of intracellular hyphae that could be interpreted as finely-branched arbuscules, though were previously interpreted by the author as zones of cellular lysis in a plant sampled from the same island locale^8^ as our material. Our structural evidence for glomalean mycorrhizal-like associations is also consistent with a previous report that, by contrast to other mosses investigated, the *T. lepidozioides* genome includes all GRAS [Gibberellic acid insensitive (GAI), Repressor of GAI (RGA), and Scarecrow (SCR)] transcription factor genes necessary to establish such symbioses, as do various hornworts, liverworts and vascular plants^5,23^. Our new metagenomic sequence data also support the concept that *T. lepidozioides* has glomalean and other mycorrhiza-like fungal associates, as well as beneficial bacterial taxa, including known nitrogen-fixers.

### Metagenomic contigs containing ribosomal RNA genes indicated a diverse microbiota of bacteria and fungi

Metagenomic sequence data consisted of 118,045,461 raw reads having maximum length of 151 base pairs (bp). These reads assembled into 1,859,383 contigs (minimal length 200 bp, maximum 172,642 bp, average 566, N50 544 bp). Total assembly length was 1,053,462,731 bp. We employed contigs to achieve greater taxonomic confidence, since longer sequences had to meet specified identity criteria^11^ (Supplementary Tables 1, 2). Contig-based taxonomy yielded 40 bacterial generic or species determinations based on 16S rRNA gene sequences, and 52 based on the 23S rRNA gene. Contig-based analysis indicated at least 18 fungal genera or species based on 18S rRNA gene sequences, 13 based on 28S rRNA genes, and 17 from ITS. The assembly produced 15 contigs that contained ITS regions; some with full ITS (both ITS1 and ITS2), and the rest either ITS1 or ITS2 (Supplementary Table 3). We note that annotation results based on ITS1 or ITS2 were sometimes different than those based on full ITS sequences, and annotation results from searching against the UNITE database sometimes differed from those found by searching the GenBank nr database. Many contigs and unassembled reads (Supplementary Tables 4, 5) were classifiable only at high taxonomic levels, indicating the presence of new diversity.

### Metagenomic data confirm that *T. lepidozioides* hosts mycorrhiza-like fungal species

Mycorrhizal fungi are known to provide diverse land plants with mineral nutrients essential to reducing growth limitation, but are thought to be absent from mosses^2,23^. However, a focused search of our metagenomic assembly for glomalean fungi revealed four contigs indicating presence of glomalean fungi at high genomic coverage levels, two of which may represent undescribed taxa (Table 1). High genomic coverage levels may reflect large numbers of nuclei typical for glomalean spores. In addition, contig analysis of both 18S and 28S rRNA gene sequences indicated presence in the *T. lepidozioides* microbiome of the ascomycete *Leotia lubrica*, a helotialean fungal species known to associate with plants to form ericoid mycorrhizae^2^ (see Supplementary Tables 1, 2). A contig containing the 18S rRNA gene marker also indicated the ascomycete *Rhizoscyphus ericae* (= *Hyaloscypha hepaticola*), which is known to form mycorrhiza-like associations with liverworts^23^ (see Supplementary Table 2). Based on phylogenetic position as the extant land plant closest to the divergence of bryophytes from the clade encompassing modern vascular plants^1^, at least some of the mycorrhiza-like fungi we observed to be associated with archaic *Takakia* may reflect nutrient-providing fungal associations of early land plants, including early vascular plants.

**Table 1:** *Takakia lepidozioides* metagenomic contigs classifying as glomalean fungi.

Contig number	Classification (GenBank accession)	Average genomic coverage	Genomic coverage standard deviation
K141_1305061	*Rhizophagus irregularis* (XM_025309795.1)	5120.17	1848.06
K141_704588	*Racocetra castanea* = *Scutellospora castanea* (AF038590.1), *Gigaspora gigantea* (MW739973.1), or *Gigaspora margarita* (MW739971.1)	5222.48	3064.09
K141_4888592	Uncultured glomeraceous AM fungus (AY903747.1)	6257.54	3091.06
K141_368293	Glomeromycotina sp. (MG829418.1)	5445.53	2561.46

### The *T. lepidozioides* microbiome includes bacteria and genes associated with N-fixation

**Figure 3:**
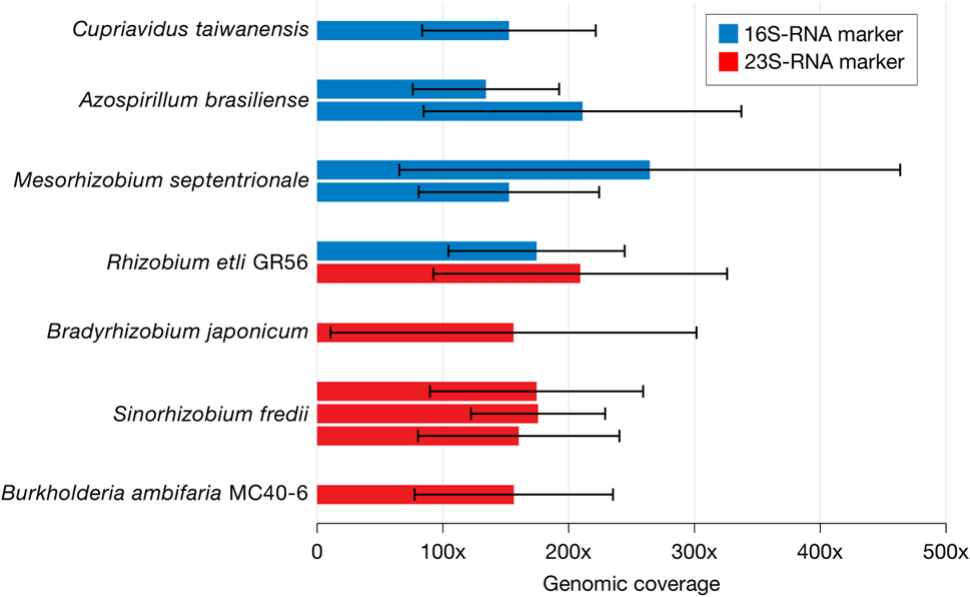
Abundances of nitrogen-fixing bacterial species in the *Takakia* microbiome. Population abundances indicated by mean genomic coverage in excess of 130X indicate that several bacterial species capable of nitrogen fixation may have been attracted by host exudates, as *T. lepidozioides* is known to produce diverse flavonoids^24^. One standard deviation shown, indicating degree of variance among the base pairs (N) of a contig in the degree to which each base pair matched relevant raw read sequences. Some of these species were detected only by the 16S rRNA gene marker, and others only by the 23S rRNA gene marker; *Rhizobium etli* GR56 was detected by both taxonomic markers. Presence of 2–3 bars represent different contigs classifying to these taxa.

The *Takakia lepidozioides* metagenome indicated multiple types of bacteria known for nitrogen fixation capacity whose ribosomal RNA genes were present at high genomic coverage (mean > 130X) (Fig. 3), and that were also represented by nifH protein coding genes commonly used as markers of N-fixation. 16S rDNA gene taxonomy indicated presence of three N-fixing bacterial species: the nodulator *Cupriavidus taiwanensis* indicated by a 289 bp contig having 100% sequence identity to database reference sequences; the nodulator *Mesorhizobium septentrionale* inferred from two contigs, including a 774 bp sequence of > 98% identity; and free-living *Azospirillum brasilense* indicated by a 830 bp contig of > 98% similarity. 23S rDNA gene taxonomy indicated the presence of additional N-fixing bacterial species: the nodulator *Bradyrhizobium japonicum* inferred from a 483 bp contig of > 97% similarity; *Rhizobium etli* GR56 inferred from a 663 bp contig having > 98% similarity; *Sinorhizobium fredii*, a nodulator having notably wide host range, indicated by several contigs, including a 533 bp contig having 100% similarity; and *Burkholderia ambifaria* MC40-6 inferred from a 230 bp contig of 100% similarity. *Takakia* species are known to produce a variety of flavonoids^24^, important because higher plants employ flavonoid signals to attract nitrogen-fixing bacteria during early stages of root nodule development. Presence of nitrogen-fixing bacteria was also supported by *nifH*-related sequences.

Six contigs from our metagenomic assembly included partial sequences of a gene encoding nitrogenase iron protein (nifH) (Supplementary Tables 6, 7, 8, 9, 10, 11). The nodulator species *Cupriavidus taiwanensis*, *Bradyrhizobium japonicum* and *Sinorhizobium fredii* were represented by nifH-related protein-coding sequences, as were *Azospirillum*, *Rhizobium*, *Mesorhizobium* and *Burkholderia* and other bacterial genera. We did not find significant ribosomal or *nifH* marker evidence for the presence of cyanobacteria in the *T. lepidozioides* microbiome; none was detected by > 10 unassembled reads or by any contig classification or *nifH* data. Given the ubiquity of cyanobacteria, and their abundance in moist environments, this low cyanobacterial signal provides evidence that the washing procedures we employed to clean *Takakia* prior to DNA extraction were effective in removing all but closely-associated microbes.

Metagenomic evidence for genus *Rhizobium* in the *T. lepidozioides* microbiome–indicated by both ribosomal and *nifH* marker genes–was shared with aquatic Coleochaetales streptophyte algae and rock-dwelling *Conocephalum conicum* (liverwort) previously studied using similar preparation methods^14^. *Bradyrhizobium* was observed to be a relatively-abundant associate shared with *Sphagnum fimbriatum*^15^, representing Sphagnales, which are close moss relatives of Takakiales^1^. These commonalities suggest that N-fixing bacterial associations likely aided plant N-acquisition long before the evolution of root nodules in angiosperms.

### The *T. lepidozioides* microbiome includes bacterial and fungal associates that otherwise promote plant growth

As was the case for other bryophytes and streptophyte algae studied similarly^14,15^, the *T. lepidozioides* microbiome included microbial species known to promote plant growth. *Burkholderia phytofirmans*, detected by 279 bp of 23S rRNA gene sequence that was 100% similar to database references, is a known plant growth promoter, as is *Variovorax paradoxa* S110, indicated by 302 bp of 23S rRNA gene sequence that was 99.8% similar to references. *Janthinobacterium* sp. 4239 was indicated by a 365 bp sequence of 16S rRNA gene having 100% identity to database references; species of this taxon are known for producing antifungal compounds^25^. Likewise, *Collimonas fungivorans* was indicated by 336 bp of 23S rRNA gene having 100% similarity to database references, suggesting chitinolytic, anti-fungal protective functions^26^. The single most abundant bacterial species, detected by 1056 SSU and 1758 LSU unassembled reads, was *Acidobacterium capsulatum*; some strains of Acidobacteria are known to promote plant growth, possibly fostering root development by producing auxins^27^. Likewise, abundance of the acidobacterium Candidatus *Koribacter versatilis* indicated by 1211 unassembled LSU reads indicates importance to *Takakia* in some way.

The nematophagous fungus, *Mortierella globalpina* (Mucoromycota) was indicated by a 752 bp sequence of the 28S rRNA gene having > 97% identity to database references. *Trichoderma viride*, whose hyphal coils degrade plant pathogen cell walls and so has been advocated as a biocontrol agent^28^, was detected by a 380 bp sequence of 18S rRNA gene and ITS sequence. Entomopathogenic *Varicosporium elodea* (indicated only by ITS) may help protect against insect damage.

### The *T. lepidozioides* microbiome includes protists and evidence from small animals

A 1190 bp 28S contig had 100% sequence identity to *Cosmarium punctulatum* Brébisson^29^, a streptophyte algal species. This identification may be consistent with presence of a morphologically similar structure within the mucilage cloud of a mucilage organ (see Fig. 1f). Multiple contigs indicated presence of the arthropod springtail *Pogonognathellus flavescens*; springtails are known to inhabit mosses, where they can facilitate sexual reproduction^30^.

### Implications for understanding early land plant microbiomes

Based on its phylogenetic position among extant plants, the early-diverging moss *Takakia* can be viewed as a model of early land plants, providing information about symbiosis-related aspects of the evolutionary transition from streptophyte algae to embryophytes. Structural and microbiome commonalities with streptophyte algae that likewise maintain microbiota within conspicuous envelopes of secreted mucilage indicate possible co-option by early land plants of ancestral algal symbiosis adaptations. This first metagenomic study of the *Takakia* microbiome identified several types of abundant N-fixing bacteria, additional bacterial species that may benefit plant growth, and several types of mycorrhiza-like fungal species, the latter previously thought to be absent from mosses. Assuming that the *T. lepidozioides* microbiome at least partially mirrors that of early land plants, these observations indicate symbiosis adaptations to nutrient-poor early soils.

**Figure 4:**
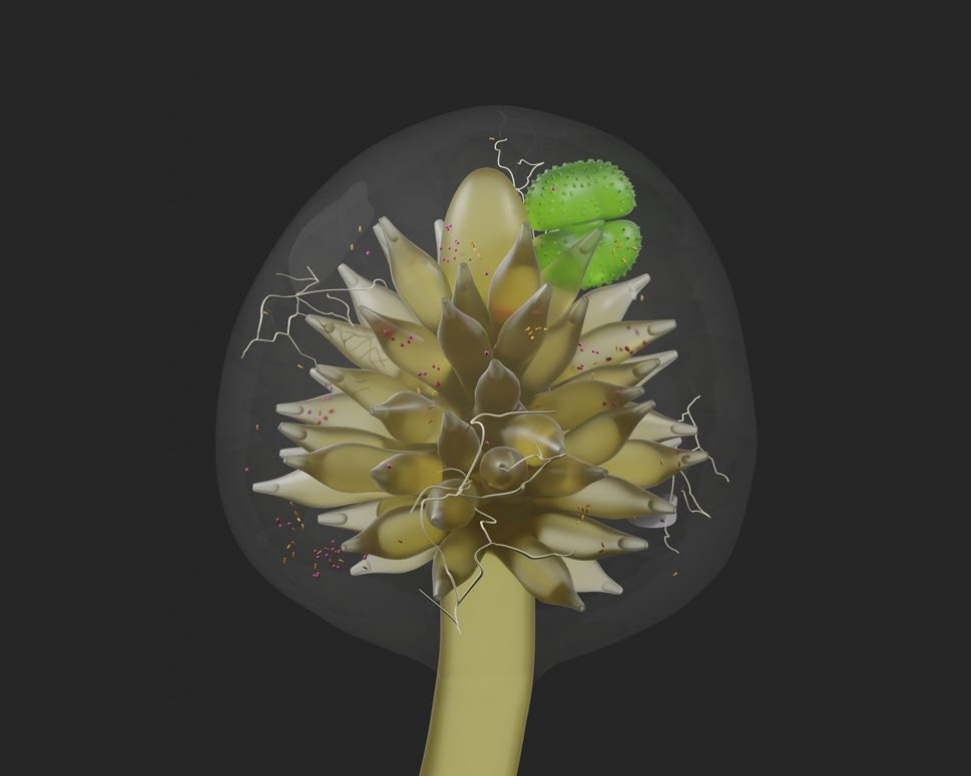
Model of microbial associations with the mature *Takakia* mucilage organ. A three-dimensional model of the mature *T. lepidozioides* mucilage organ illustrates its role in amplifying the production of mucilage that harbors abundant and diverse microbial associates, including bacteria, fungi, and other microeukaryotes. Microbes are also depicted as occupying mucilage within open-tipped mucilage cells. Evidence for endogenous origin of the core parenchymatous lateral axis from a rhizomatous (root-analog) axis, presence of an apical meristem (top), and intracellular location of microbes are features suggesting analogies to specialized lateral roots of angiosperms that are adapted in ways that foster beneficial microbial associations. These features also indicate that dehisced mucilage organs might be able to function as asexual reproductive structures that transmit microbial associations from one generation to the next.

We discovered that *Takakia* rhizomatous (root-analog) axes produce a previously-unrecognized type of lateral organ that amplifies the production of mucilage that harbors microbiota (Fig. 4), analogous to higher plant lateral root specializations fostering N-fixing symbionts. The presence of apical meristematic tissues suggests that *Takakia* mucilage organs may have the potential for elongation into lateral “roots” whose bases are ringed by mucilage cells, as well as potential for dispersal and regeneration of young plants, as has been suggested for related *T. ceratophylla*^31^. Dispersal of asexual propagules in the form of mucilage organs would benefit *T. lepidozioides*, since North American populations of this species (though known to produce archegonia) are not known to produce sporophytes, so may not be able to disperse by means of meiospores. If early land plants more often reproduced asexually, such plants might not have left abundant sexually-produced spores in the fossil record, a prospect that might help to explain a time gap between the origin of land plants indicted by molecular diversification studies and the first records of spores that are confidently attributed to land plants. Dispersal of mucilage organs would also benefit early plants by providing derivative young plants with beneficial microbiota, allowing serial microbiome transfer from one generation to the next. Future studies of modern *Takakia*-microbial associations might provide additional useful information, but depend on conserving rare *Takakia* habitat, which likely includes previously unrecognized remote locales. For example, our previous metagenomic study of remote Southern Patagonian moss populations yielded evidence that *Takakia*, not previously reported from South America, was present^15^.

## Conclusions

This report describes symbiosis-related metagenomic and structural features that illuminate the evolutionary transition from streptophyte algae to early embryophytes, represented by the early-diverging moss *Takakia lepidoziodes*. We provide an additional record for the *T. lepidozioides* plastid genome and show that this moss shares with streptophyte algae secretion of microbe-harboring mucilage and bacterial taxa and genes indicating nitrogen fixation. We document that *Takaki*a root-analogs produce lateral mucilage organs that are more complex than generally understood, having structural analogies to angiosperm lateral roots adapted for N-fixation symbioses. We also report structural and metagenomic evidence for mycorrhiza-like species of glomalean and ascomycete fungi not previously known for mosses. Because *Takakia* is the oldest known modern plant genus, our metagenomic study of carefully washed plants from a remote locale not strongly influenced by human activities may indicate microbiome features of early land plants.

## Material and methods

### Plant material

#### Collection information

In June 2018, *T. lepidozioides* was collected using aseptic methods, and under permit, by G. K. Golinski from Takakia Lake, Moresby Island, Haida Gwaii, British Columbia, Canada (52°55′48″N, 132°04′15″W). At this coastal northern temperate rain forest site, the average high temperature during the month of collection is 10°C, and the average low temperature 4°C. A fist-sized clump dominated by this moss that included a few thalloid liverworts and angiosperm seedlings but not soil, was stored over damp paper towels in a clear plastic box (allowing light penetration) in a lighted refrigerator in the University of British Columbia Herbarium for a month prior to cleaning and processing for subsequent DNA extraction or microscopy. This storage regime might have influenced the relative abundances of microbes indicated by DNA extracted a month later, but was designed to reduce the chances of microbial contamination or overgrowth during this period, and by maintaining conditions conducive for plant survival, to reduce the chances for loss of key microbial associates.

#### Cleaning and chemical-stabilization processes

Individual *Takakia* plants were isolated from less common plants with the use of sterile fine forceps and a dissecting microscope, employing the inner surface of a clear sampling bag (Whirl–Pak) as a sterile operating field. Several plants were placed into each of ~ 50 sterile 1 ml Eppendorf tubes containing sterilized mineral growth medium (DYIII)^32^ to maintain osmotic conditions, then gently agitated to remove loosely attached materials. The washing liquid was removed using a sterile pipette, then discarded, and the washing process repeated twice more. Following this cleaning process, which mirrored those we had previously used to clean streptophyte algae and other seedless plants prior to metagenomic analysis^13–15^, plants were stored in 1 ml Eppendorf tubes in Qiagen RNAlater (RNAprotect) for later DNA extraction, or in 2% EM-grade glutaraldehyde freshly prepared in pH 7 0.1 M phosphate buffer, for later microscopy. After two hours, glutaraldehyde fixed plants were washed three times in phosphate buffer, and stored in buffer for transport. Capped tubes were stored for a few days in a refrigerator until air transport to the US at ambient temperature, then glutaraldehyde-fixed materials were stored in a refrigerator and chemically-stabilized materials for DNA studies were stored in a -20 freezer.

### Microscopy

Some glutaraldehyde-fixed plants were stained with methylene blue to enhance detection of mucilage and microbes contained therein^7^. Other glutaraldehyde-fixed material was prepared for semi-thin sectioning by washing three times with distilled water prior to post-fixation with freshly-prepared aqueous 1% osmium tetroxide for two hours, then dehydration in an ethanol series and embedment in LR White acrylic resin (Sigma-Aldrich, St. Louis, MO, USA). Semi-thin sections cut using a Sorvall MT2B ultramicrotome and DuPont diamond knife were collected from a water surface using ethanol-cleaned copper wire loops for transfer to glass slides, then sections were stained with a solution of 1% toluidine blue + 1% sodium borate and embedded in Permount (Thermo-Fisher, Fitchburg, WI, USA) to generate permanent slide mounts. Stained sections were used as the basis for three-dimensional reconstruction of an entire mucilage organ showing the locales of associated microbes of diverse morphotypes.

Whole plants treated with concentrated acetic acid for at least 24 h were examined using a Zeiss Axioplan fluorescence microscope equipped with UV (G365 FT395 LP420) and violet (395–440 FT460 LP470) filter sets for evidence of cell wall fluorescence properties previously linked to hydrolysis-resistance in streptophyte algae^21^ and peat mosses that are closely related to *Takakia*^22^. Images were recorded using a Nikon D300s digital camera and Camera Control Pro software (Nikon, Melville, NY, USA) or an Olympus BX50 compound microscope equipped with Nikon D810 camera.

### DNA extraction, metagenomic sequencing, and informatic processing

DNA extraction was performed using the MoBio PowerSoil kit (Qiagen, Hilden, Germany) employing a modified lysis procedure designed to reduce DNA shearing, as suggested by Qiagen technical personnel: samples were vortexed for only a few seconds prior to heating at 70 °C for 5 min, a process that was then repeated. DNA from washed plant material in several Eppendorf tubes was pooled to provide sufficient DNA for metagenomic sequencing, which was done at the University of Wisconsin Biotechnology Center using the Illumina HiSeq 2500 platform. Raw reads were trimmed using Trimmomatic version 0.39 using SLIDING WINDOW:4:30^33^. MEGAHIT version 1.2.9, designed for handling large, complex metagenomics data sets^34^, was employed for assembling contigs from raw sequences. The assembler used paired reads to detect and resolve chimeric contigs produced from misassembly^35^.

Molecular taxonomy using contigs as input to the taxonomic platform provided more-conservative identifications by comparing longer sequences^11^. The MG-RAST metagenome analysis server^36^ was used to classify both contigs and short reads containing 16S/18S rDNA and 23S/28S rDNA. The pipeline of MG-RAST searched all sequences in the assembly for potentially rRNA genes with a cut-off of 70% identity to ribosomal sequences from a reduced version of M5RNA. Then, to re-filter the chimeric sequences from the MG-RAST results, we used VSEARCH version 2.8.3.0^37^ implemented in Galaxy platform^38^ to search all sequences annotated as rDNA obtained from MG-RAST results against the SILVA SSU and LSU RefNR99 release 138^39^. The parameters were minh—minimum score to report chimera = 0.3, mindiv—minimum divergence ratio = 0.5, xn—weight of a no vote = 8, dn—pseudo-count prior on number of no votes = 1.4, minimum number of differences in segment = 3. Finally, annotation of these non-chimeric rDNA sequences was done by searching against SILVA SSU and LSU databases implemented in the MG-RAST pipeline using threshold of E-value = 1E-5, percent identity = 97, minimum length = 15. The final rDNA sequences and their annotations are located in Supplementary Tables 1 and 2. For calculation of genomic coverage displayed in Supplementary Tables 1 and 2, trimmed raw reads were aligned back to the contigs with annotated rDNA using BWA version 0.7.4 non-model species alignment^40^. SAMtools version 1.7 was used to convert the output SAM file to sorted BAM file and bedtools version 2.26.0^41^ was used to find the coverage of every position of the contigs, with mismatches not allowed. For each contig, some regions aligned to more short reads than did others, so the mean number of aligned short reads and one standard deviation of variance were manually calculated for each contig. N therefore equaled the number of base pairs in a contig.

For completeness, we also performed analyses using unassembled read sequence data. Trimmed raw reads were submitted for taxonomic identification to the MG-RAST metagenome analysis server, which classified 16S/18S and 23S/28S rRNA genes using a cut-off of 70% identity to ribosomal sequences from a reduced version of M5RNA. Then, annotations of rRNA gene reads were obtained by searching against the SILVA SSU and LSU databases implemented in the MG-RAST pipeline using a threshold of E-value = 1E-5, percent identity = 97, minimum length = 15.

To focus on fungi we employed ITS regions and the UNITE database. We used ITSx version 1.1.3^42,43^ to search metagenomic sequences for nuclear ribosomal internal transcribed spacers (ITS). This Perl-based software tool extracts ITS1, 5.8S, ITS2, and full-length ITS sequences by searching the assembled metagenomic fasta file against the HMMs of organisms using HMMER version 3.3.2 for profile hidden Markov model analysis^44^. Because results were mostly unknown taxa, BLASTN was then used to search sequences against the GenBank nucleotide database using blastn suite server available at https://blast.ncbi.nlm.nih.gov/Blast.cgi (accessed on July 13, 2021).

To focus on glomalean fungi indicated by microscopic observations, we downloaded all the nucleotide sequences from glomalean fungi available in the GenBank database (accessed on July 21, 2021). Then we used the GenBank glomalean sequences as queries to search against our metagenomic assembly using a local BLASTN version 2.6.0 + with the threshold of E-value = 1E-10. Then the search results, which included contigs from the assembly, were used as queries to search against the NCBI nucleotide nr/nt database (accessed on July 22, 2021). For calculation of genomic coverage of contigs linked to glomaleans, trimmed raw reads were aligned back to the contigs with annotated rDNA using the BWA version 0.7.4 non-model species alignment by allowing mismatch (−M) = 0^40^. SAMtools version 1.7 was used to convert the output SAM file to a sorted BAM file, and bedtools version 2.26.0^41^ was employed to find the coverage of every base position of the contigs, then contig mean coverage and standard deviations were manually calculated.

Taxa inferred from rRNA gene sequences at a mean coverage level > 10X were considered to have been detected and employed for evolutionary comparisons to those we detected in microbiomes of other seedless plants and streptophyte algae we had studied similarly^13–15^. Because the sequence data were obtained by shotgun metagenomic methods, amplification bias was not an issue, as can be the case when input sequences are amplicons. However, we note that for eukaryotes, sequencing depth levels may be influenced by multicellularity or genomic duplication history. In all cases, identifications at the species level should not be considered completely accurate unless the percent identity = 100 and alignment length covers the entire ribosomal gene.

To focus on nifH protein-coding sequences, commonly used as a marker of nitrogen fixation, we used the bacterial UniProtKB/Swiss-Prot protein sequences available to the GenBank database (accessed on July 25, 2021) as queries to search against our metagenomic assembly using a local BLASTN version 2.6.0 + with the threshold of E-value = 1E-10. Then search results, which included contigs from our assembly, were used as queries to search against the NCBI non-redundant protein sequences (nr) database (accessed on July 25, 2021).

We also used trimmed raw reads to assemble the *Takakia lepidozioides* plastid genome using MEGAHIT version 1.2.9^34^, using the parameter “bubble-level equal to 0” in order to prevent the merging of sequences that were highly similar, e.g., sequences from closely related species or sequences that display single nucleotide polymorphisms. The protein-coding genes were annotated using proteins inferred from the unpublished *T. lepidozioides* chloroplast (NC_028738.1) and *Sphagnum palustre* (NC_030198.1)^45^. The tRNAs and rRNA genes were annotated using tRNAscan-SE On-line^41^ and RNAmmer 1.2 Server^46,47^.

## Supplementary Information

Supplementary Figure 1.

Supplementary Table 1.

Supplementary Table 2.

Supplementary Table 3.

Supplementary Table 4.

Supplementary Table 5.

Supplementary Table 6.

Supplementary Table 7.

Supplementary Table 8.

Supplementary Table 9.

Supplementary Table 10.

Supplementary Table 11.

## Data Availability

Raw metagenomic sequences were deposited in the NCBI SRA as BioProject PRJNA754003, BioSample SAMN20720473: *Takakia lepidozioides* Takakia Lake British Columbia (TaxID: 1,297,885), SRA: SRR15431198. The MG-RAST IDs for the selected assembled contigs and short reads were mgm4936586.3 and mgm4936670.3, respectively. The complete plastid genome of Takakia Lake *Takakia lepidozioides* was deposited in GenBank accession number MZ895084.
