# Supplementary figures and images for: Microbiome and related structural features of Earth’s most archaic plant indicate early plant symbiosis attributes
Source: Sci Rep. 2022 Apr 20;12:6423. doi: 10.1038/s41598-022-10186-z (PMC9021317; doi:10.1038/s41598-022-10186-z)

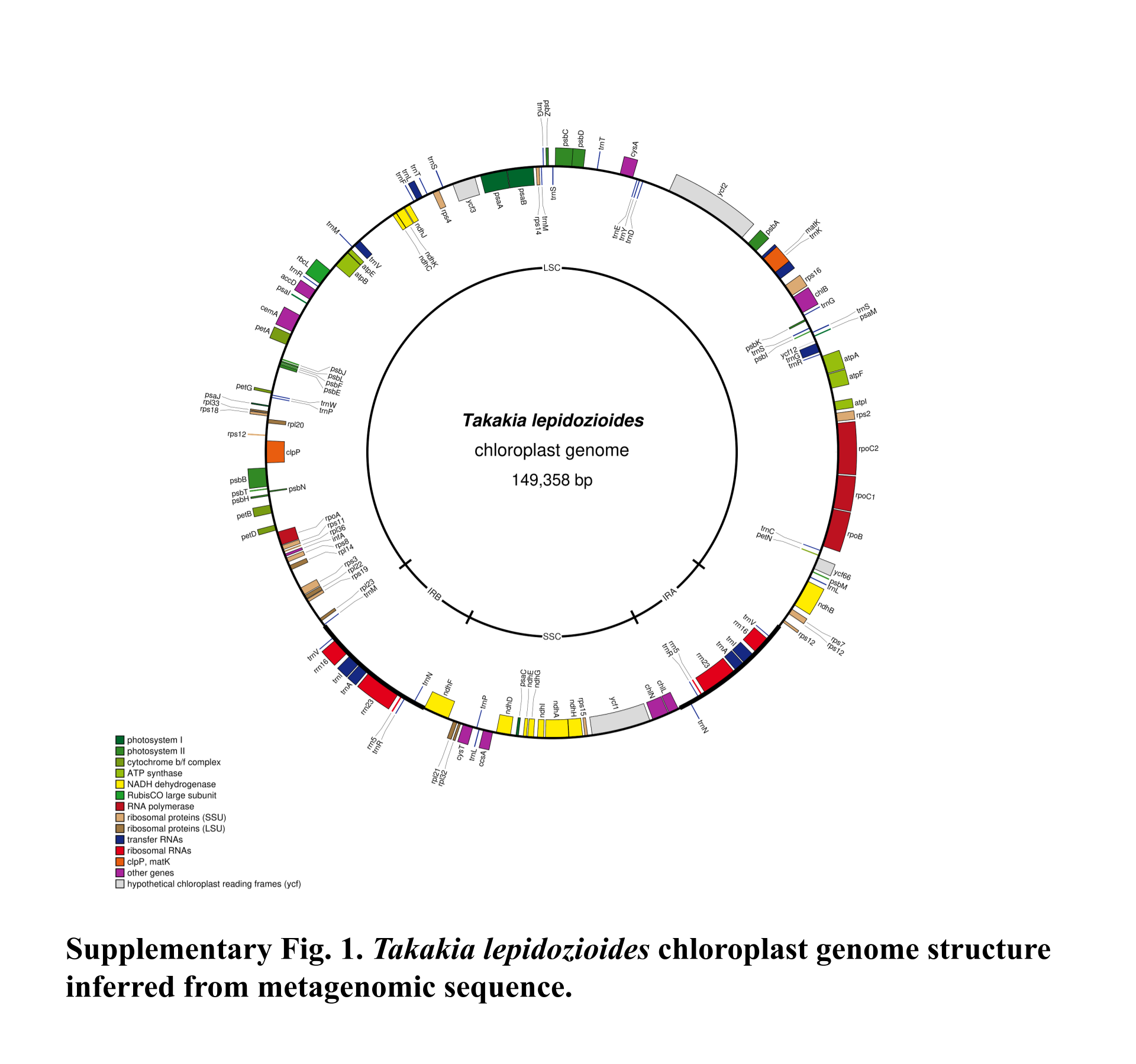

Supplement: Supplementary file 1 — Supplementary Figure 1. [file 41598_2022_10186_MOESM1_ESM.png]
