# Supplementary material for: Microbiome and related structural features of Earth’s most archaic plant indicate early plant symbiosis attributes
Source: Sci Rep. 2022 Apr 20;12:6423. doi: 10.1038/s41598-022-10186-z (PMC9021317; doi:10.1038/s41598-022-10186-z)
Supplement: Supplementary file 4 — Supplementary Table 3. [file 41598_2022_10186_MOESM4_ESM.docx]

Supplementary Table 3. Fungal and other eukaryotic annotation results based on the UNITE database and GenBank nt database, together with genomic coverage estimates. Note that genomic coverage is less reasonable as an estimate of organismal abundance for eukaryotes than for prokaryotic taxa, given the occurrence of multicellularity and genomic duplication events common in the ancestry of eukaryotes.

| No. | Summary |
| --- | --- |
| 1 | Contig name: k141_938853 flag=0 multi=6.1561 len=1089  Coverage: 570.68 ± 394.55  ITS1 present  UNITE annotation: Envir Eukaryota  BLASTN results: uncultured fungi and fungi belonging to Hyaloscyphaceae  ITS2 present  UNITE annotation: Envir Eukaryota  BLASTN results: uncultured fungus, Hyaloscyphaceae sp., and *Hyphodiscus* sp.  Full ITS present  UNITE annotation: Helotiales  BLASTN results: uncultured endophytic fungi and Helotiales species  Note: matches with bryophytic endophytic fungi (GenBank JX852365), Antarctica |
| 2. | Contig name: k141_1182442 flag=1 multi=3.0000 len=1130  Coverage: 702.70 ± 773.46  ITS1 present  UNITE annotation: Envir Eukaryota  BLASTN results: uncultured metazoan and *Tomocerus jesonicus*, *Tomocerus varius*, *Tomocerina liliputanus*, and *Tomocerus* sp.  ITS2 present  UNITE annotation: Envir Eukaryota  BLASTN results: *Tomocerus jesonicus*, *Tomocerus varius*, *Tomocerina liliputanus*, *Tomocerus* sp. KHP-2008, and *Pogonognathellus flavescens*.  Full ITS not present |
| 3. | 3. k141_1651015 flag=0 multi=4.7054 len=528  Coverage: 576.00 ± 500.24    ITS1 present  UNITE annotation: Helotiales  BLASTN results: uncultured Helotiales and uncultured Dermateaceae  Note: match to non-ectomycorrhizal Helotiales froma study of fungi associated with living parts of boreal forest bryophytes  ITS2 not present  Full ITS not present |
| 4. | 4. k141_984392 flag=1 multi=2.0000 len=528  Coverage: 831.05 ± 1,193.35  ITS1 present  UNITE annotation:  BLASTN results: uncultured *Pezizomycotina*  ITS2 not present  Full ITS not present |
| 5. | 5. k141_990899 flag=1 multi=4.2184 len=2472  Coverage: 736.86 ± 468.45  ITS1 present  UNITE annotation: Envir Eukaryota  BLASTN results: *Trichoderma asperellum*, *Epichloe typhina*, *Epichloe festucae var. Lolii*, *Epichloe hybrida*, and *Nectria cinnabarina*.  ITS2 present  UNITE annotation: Envir *Trichoderma*  BLASTN results: *Trichoderma asperellum*, *Epichloe typhina*, *Epichloe festucae var. lolii,* *Epichloe hybrida*, and *Nectria cinnabarina*.  Full ITS present  UNITE annotation: *Trichoderma viride*  BLASTN results: *Trichoderma viride* |
| 6. | 6. k141_310628 flag=0 multi=3.4035 len=1648  Coverage: 573.83 ± 287.55  ITS1 present  UNITE annotation: Envir Eukaryota  BLASTN results: uncultured fungus, *Cladophialophora minutissima*, *Cladophialophora sylvestris*, *Chaetothyriales* sp. EF801, and *Chaetothyriomycetidae* sp. NC1418  ITS2 present  UNITE annotation: Chaetothyriales  BLASTN results: uncultured fungi  Full ITS present  UNITE annotation: Envir Eukaryota  BLASTN results: *Cladophialophora minutissima*  Note: a number of matches with studies of bryophytes in Antarctica |
| 7. | 7. k141_1529372 flag=1 multi=96.7119 len=21667  Coverage: 944.58 ± 778.67  ITS1 present  UNITE annotation: unidentifiable  BLASTN results: *Takakia lepidozioides* and some other bryophytes  ITS2 present  UNITE annotation: unidentifiable  BLASTN results: *Takakia lepidozioides* and some other bryophytes  Full ITS present  UNITE annotation: Envir Bryophyta  BLASTN results: *Polytrichum piliferum* |
| 8. | 8. k141_1099417 flag=0 multi=5.4271 len=525  Coverage: 486.50 ± 252.90    ITS1 present  UNITE annotation: Envir Eukaryota  BLASTN results: uncultured Ascomycota, *Fontanospora fusiramosa*, and *Articulospora tetracladia*.  ITS2 not present  Full ITS not present |
| 9. | 9. k141_395727 flag=0 multi=3.0607 len=1657  Coverage: 691.70 ± 490.49  ITS1 present  UNITE annotation: Mycosphaerellaceae  BLASTN results: *Acrodontium crateriforme*, *Neocatenulostroma microsporum*, *Teratosphaeria destructans*, *Pseudocercospora ocimicola*, and *Teratosphaeria molleriana*.  ITS2 present  UNITE annotation: Fungi  BLASTN results: *Acrodontium crateriforme*, *Neocatenulostroma microsporum*, *Teratosphaeria destructans*, *Pseudocercospora ocimicola*, and *Teratosphaeria molleriana*.  Full ITS present  UNITE annotation: Mycosphaerellaceae  BLASTN results: uncultured *Acrodontium* and *Acrodontium crateriforme* |
| 10. | 10. k141_1816615 flag=0 multi=1.0000 len=335  Coverage: 502.64 ± 330.25  ITS1 present  UNITE annotation:Helotiaceae  BLASTN results: *Pezoloma ericae*  ITS2 not present  Full ITS not present |
| 11. | 11. k141_1816618 flag=1 multi=0.9321 len=303  Coverage: 515.22 ± 293.06  ITS1 present  UNITE annotation: Fungi  BLASTN results: uncultured Ascomycota, *Herpotrichiellaceae* sp. Sig1, and *Cladophialophora* sp.  Note: similar to #6 above.  ITS2 not present  Full ITS not present |
| 12. | 12. k141_559682 flag=0 multi=1.0000 len=635  Coverage: 569.06 ± 354.32  ITS1 not present  ITS2 present  UNITE annotation: *Fontanospora fusiramosa*  BLASTN results: uncultured fungi, *Articulospora tetracladia*, and *Fontanospora eccentrica*, Helotiales, *Gyoerffyella*, *Tricladium*  Full ITS not present |
| 13. | 13. k141_1050707 flag=1 multi=2.0000 len=671  Coverage: 770.40 ± 354.06    ITS1 not present    ITS2 present  UNITE annotation: Envir Viridiplantae  BLASTN results: *Epigaea repens*, *Rhododendron lapponicum*, *Oxydendrum arboreum*, *Kalmiopsis leachiana*, and *Empetrum nigrum*.  Full ITS not present |
| 14. | 14. k141_1074226 flag=1 multi=2.0000 len=306  Coverage: 664.59 ± 437.11    ITS1 not present  ITS2 present  UNITE annotation: Fungi  BLASTN results: fungal sp., uncultured Pleosporales, Pleosporales sp., and *Phoma* sp.  Full ITS not present |
| 15. | 15. k141_1113822 flag=1 multi=1.0000 len=341  Coverage: 641.16 ± 711.60  ITS1 not present    ITS2 present  UNITE annotation: Fungi, Sclerotiniaceae, Helotiales  BLASTN results: *Botryotinia ranunculi* and *Botrytis cinerea*.  Full ITS not present |
